# Five blueberry anthocyanins and their antioxidant, hypoglycemic, and hypolipidemic effects *in vitro*

**DOI:** 10.3389/fnut.2023.1172982

**Published:** 2023-05-18

**Authors:** Chao-wei Zhu, Han Lü, Lan-lan Du, Jing Li, Han Chen, Hui-fang Zhao, Wen-long Wu, Jian Chen, Wei-lin Li

**Affiliations:** ^1^Department of Food Science and Technology, College of Light Industry and Food Engineering, Nanjing Forestry University, Nanjing, China; ^2^Jiangsu Key Laboratory for the Research and Utilization of Plant Resources, Institute of Botany, Jiangsu Province and Chinese Academy of Sciences, Nanjing, China; ^3^Co-Innovation Center for Sustainable Forestry in Southern China, Forestry College, Nanjing Forestry University, Nanjing, China

**Keywords:** blueberry anthocyanin, diabesity, antioxidant, hypoglycemic, hypolipidemic

## Abstract

The dual epidemic of obesity and diabetes mellitus is becoming an important worldwide public health issue. “Diabesity” is the term used to describe the combined detrimental health effects of both diabetes mellitus and obesity/overweight. Currently, food-derived bioactive compounds are suggested to alleviate diabesity. Blueberries are rich in bioactive anthocyanins, which are associated with contributing to preventing obesity and diabetes mellitus. However, the accurate active compounds and the underlying mechanism are still unclear. The objective of this study was to investigate the beneficial effects of blueberry anthocyanin on diabesity. In total, five anthocyanins (delphinidin-3-*O*-galactoside, delphinidin-3-*O*-glucoside, petunidin-3-*O*-galactoside, petunidin-3-*O*-glucoside, and malvidin-3-*O*-galactoside) were isolated from rabbiteye blueberry (*Vaccinium virgatum*) cultivar “Garden blue.” All these anthocyanins exhibited oxygen radical absorbance capacity (ORAC), scavenging power of ABTS^+^, and DPPH-free radical and inhibitory activity of α-glucosidase *in vitro*. Moreover, some compounds improved glucose uptake and attenuated lipid accumulation in high glucose and oleic acid-treated HepG2 cells. All these results suggest that blueberry anthocyanins have potential antioxidant, hypoglycemic, and hypolipidemic effects, which may benefit the treatment of diabesity.

## Introduction

The dual epidemic of obesity and diabetes mellitus is becoming an important worldwide public health issue. The prevalence of obesity has been on the rise in the past decades. By 2030, it is predicted by the WHO that 38% of adults worldwide will be overweight and 20% of them will be obese (1). Increased adiposity, which is frequently associated with multiple chronic comorbidities, is looked at as the strongest risk factor for developing type 2 diabetes mellitus (T2DM) (2). According to the International Diabetes Federation (IDF), the global diabetic patients aged 20–79 years are estimated to be 536.6 million in 2021, of which T2DM accounts for approximately 90% of the total (3). “Diabesity” is a term used to describe the combined detrimental health effects of T2DM and obesity/overweight (4). The presence of diabesity enhances the risk of numerous complications including retinopathy, nephropathy, neuropathy, cardiovascular diseases, stroke, and liver diseases such as non-alcoholic fatty liver disease (5). Bariatric surgery is a therapeutic approach for diabesity that results in diabetes remission and marked weight loss. However, it is invasive, expensive, and unacceptable to many patients. Managing diabesity remains a global challenge.

Currently, food-derived bioactive compounds are suggested to alleviate diabesity for their low toxicity, and no severe adverse events correlated with pharmacotherapeutic agents. Anthocyanins are kinds of naturally occurring polyphenolic compounds widely distributed in plant-based daily diets, including fruits, vegetables, and pigmented cereals (6). Natural anthocyanins are not only natural pigments, but they can offer beneficial effects on human health. Several related studies indicated that anthocyanins might have important implications in preventing obesity and type 2 diabetes (7, 8).

Blueberry is considered to be a “super fruit” due to its abundant anthocyanins. Previous studies have shown that regular, moderate intake of blueberries and/or blueberry extracts could reduce the risk of cardiovascular disease and T2DM and improve weight maintenance and neuroprotection (9). Although blueberry anthocyanins were reported to have hypoglycemic and hypolipidemic effects in both *in vitro* and *in vivo* assays due to the lack of commercial anthocyanin standards, few studies have been carried out to investigate pure individual anthocyanin for their therapeutic effects on diabesity. The accurate active compounds and the underlying mechanism are still not clear.

Therefore, in this study, five dominant anthocyanin compounds were isolated from blueberry “garden blue.” The antioxidant properties and anti-hypoglycemic and hypolipidemic activities of these anthocyanins were tested and compared *in vitro*. The results may indicate the active compounds and the potential mechanism of blueberry for the treatment of diabesity.

## Materials and methods

### Chemical and reagents

Acetonitrile, methanol, and formic acid (HPLC grade) were obtained from Tedia Co. Inc. (Fairfield, OH, USA). Ethanol and phosphoric acid were purchased from Sinopharm Chemical Reagent Co. Ltd. (Shanghai, China). Folin-Ciocalteu's phenol reagent, 2,2-diphenyl-1-picrylhydrazyl (DPPH), 2,2′-azinobis-3-ethylbenzothiazoline-6-sulfonic acid (ABTS), α-glucosidase (from *Saccharomyces cerevisiae*), 4-nitrophenyl-α-D-glucose (*p*-NPG), and 6-hydroxy-2,5,7,8-tetramethylchroman-2-carboxylic acid (Trolox) were obtained from Sigma-Aldrich (St. Louis, MO, USA). Sodium fluorescein, 2,2′-azobis-2-amidino-propane (AAPH), metformin (Met), oleic acid (OA), and 2-[N-(7-Nitrobenz-2-oxa-1,3-diazol-4-yl)amino]-2-deoxy-D-glucose (2-NDBG) were obtained from Aladdin (Shanghai, China). The assay kits of triglycerides (TG), total cholesterol (TC), and oil-red-O staining were obtained from Jiancheng Bioengineering Institute (Nanjing, China). The BCA assay kit was obtained from Biosharp (Hefei, China). Dulbecco's modified Eagle's medium (DMEM), penicillin-streptomycin, and fetal bovine serum (FBS) were purchased from Invitrogen-Gibco (Grand Island, NY). The chemicals and reagents used in this study were all of pure analytical grade.

### Extraction and purification of blueberry anthocyanin

Rabbiteye blueberry (*Vaccinium virgatum*) cultivar “garden blue” was harvested from the Baima plantation (Nanjing, Jiangsu province, China). The taxonomic classification was carried out by Prof. Weilin Li. The voucher specimens (No. 20220715) have been deposited in the Institute of Botany, Jiangsu Province, and the Chinese Academy of Sciences, Nanjing (China). Fresh blueberry fruit (5.0 kg) was extracted by ultra-sonication with 50% ethanol containing 0.1% formic acid (v/v) at room temperature. The ethanol solutions were concentrated under a vacuum. The supernatant was obtained by centrifugation at 4°C at 10,000 rpm for 10 min and purified with AB-8 macroporous resin. After elution with pure water to be sugar-free (sulfuric acid-phenol method), the total extract of anthocyanins (BBA) was obtained by elution with 50% ethanol containing 0.1% formic acid and concentrated under vacuum.

The BBA was further purified by the octadecylsilyl (ODS) column using MeOH/H_2_O (containing 0.1% formic acid) in a gradient manner to obtain 16 fractions (Frs.1–16). The desired fractions were purified by preparative HPLC (ODS-AQ, 200 × 20 mm, 5 μm). Fr.1 was purified using acetonitrile (ACN)/H_2_O (9.5:90.5, v/v) to yield BB1 (18 mg). Fr. 3 was chromatographed using HPLC with ACN/H_2_O (10:90, v/v) to yield BB2 (13 mg). Fr. 7 was separated using ACN/H_2_O (11:89, v/v) to yield BB3 (16 mg). Frs. 11 was separated using ACN/H_2_O (12:88, v/v) to yield BB4 (25 mg) and BB5 (19 mg) successively. All the fractions were monitored by HPLC prior to NMR analysis. HR-ESI-MS data were obtained using an Agilent 6530 accurate-mass quadrupole time-of-flight system (Agilent, USA). Fractions were dissolved in CD_3_OD: CF_3_CO_2_D (9:1), and ^1^H and ^13^C NMR spectra were obtained using AVANCE III 600M Hz spectrometers (Bruker Instruments, Karlsruhe, Germany).

HPLC was performed on a Dionex Ultimate 3000 HPLC system (Thermo Fisher Scientific Inc., Germany), equipped with a quaternary solvent delivery pump, an autosampler, a DAD detector, and a Chameleon workstation. An Inertsil ODS-SP (4.6 × 250 mm, 5 μm) analytical column was used, and the column temperature was maintained at 35°C. The mobile phase consisted of acetonitrile (A) and water (containing 0.34% phosphoric acid) (B) with linear elution (0–15 min, 90–85% B; 15–30 min, 85–70% B; 30–35 min, 70–60% B; 35–36 min, 60–10% B; 36–41 min, 10% B; 41–42 min, 10–90% B, and 42–47 min, 90% B). The flow rate was 1 ml·min^−1^, and anthocyanins were detected at 520 nm.

### Measurement of antioxidant capacity

DPPH assay was determined according to the reported method (10). The tested compounds were dissolved in ethanol and diluted to a series of concentrations to be used as test solutions. A total of 100 μl 0.16 mmol/L DPPH in methanol (5 mg/100 ml) was mixed with 100 μl of the test solution. The absorbance was measured after 30 min at 517 nm. A blank sample containing 100 μl of methanol in the DPPH solution was measured. The same procedure was followed for the positive control, ascorbic acid. All samples were analyzed in triplicate at five different concentrations around the EC_50_ values.

The ABTS assay was performed as the reported method (10). Briefly, ABTS^+^ radical cation was prepared by mixing a 7 mM aqueous stock solution of ABTS^+^ and 2.6 mM potassium peroxydisulfate. The reaction mixture was stored in the dark at room temperature for 12 h prior to use. The ABTS^+^ radical cation solution was diluted with methanol to an absorbance of 0.70 ± 0.02 at 734 nm. Then, 50 μl of the test solution was added to 200 μl of the ABTS^+^ radical cation solution. After 6 min, the absorbance was measured at 734 nm. A blank sample containing ABTS^+^ solution was measured. All the samples were analyzed in triplicate at five different concentrations around the EC_50_ values.

The oxygen radical absorbance capacity (ORAC) assay was performed according to the reported method with slight modifications (11). The assay was carried out in black-walled 96-well plates. Tested compounds were dissolved in 75 mM phosphate buffer (pH 7.4). In total, 20 μl sample and 20 μl Trolox solutions (1.25, 2.5, 5, or 10 μM) were mixed with 20 μl sodium fluorescein (63.0 nM final concentration) and preincubated for 5 min at 37 °C. After then, 140 μl APPH solution (12.8 mM final concentration) was added, and the fluorescence was recorded for 60 min at excitation filter 485 nm and emission filter 535 nm, respectively. A blank sample containing 20 μl of phosphate buffer in the reaction mix was prepared and measured. The area under the curve (AUC) was calculated for each sample by integrating the relative fluorescence curve. The net AUC of the sample was calculated by subtracting the AUC of the blank. The regression equation between net AUC and series concentration of Trolox was determined to establish a standard curve, and ORAC values were expressed as μmol Trolox equivalents/μmol samples using the standard curve established previously. All samples were analyzed in triplicate.

### Assay for α-glucosidase inhibitory activity

The α-glucosidase inhibitory activity was determined as previous methods with slight modifications (12). In short, the diluted sample working solution (1 μl) was added to 3 μl of α-glucosidase (0.5 U/ml in 0.1 M sodium phosphate buffer, pH 6.9) and 156 μl of 0.1 M sodium phosphate buffer (pH 6.9). Then, the mixture was incubated at 37°C for 20 min, and 40 μl of 1.5 mM *p*-NPG (dissolved in 100 mM sodium phosphate buffer, pH 6.9) was added and incubated for 20 min. Absorbance was measured by a microplate reader (Molecular Device, Sunnyvale, CA, USA). A complete reaction mixture without a sample was used as the control. Three parallel operations were performed. All the samples were analyzed in triplicate at five different concentrations around the IC_50_ values.

### Cell culture

Human hepatocellular carcinoma (HepG2) cells were obtained from Fuheng Cell Center (Shanghai, China) and incubated in normal D-glucose DMEM supplemented with 100 IU·ml^−1^ penicillin, 0.1 mg·ml^−1^ streptomycin, and 10% (v/v) heat-inactivated FBS in an incubator (5% CO_2_ and 37°C).

### Measurement of cell viability

Cellular viability was determined by the 3-(4,5-Dimethylthiazol-2-yl)-2,5-Diphenyltet-razolium Bromide (MTT) assay (13). HepG2 cells were seeded in 96-well plates at a density of 1 × 10^4^ cells/well and incubated for 24 h. To test the cell viability effect of BB1-BB5 in HepG2 cells, the cells were treated with different concentrations (25, 50, and 100 μM) of each compound. After being treated with samples for 12 h, media was discarded and cells were incubated with the MTT solution (Sigma-Aldrich, St Louis, MO, USA, 0.5 mg/mL) for 4 h. After incubation, the MTT solution in each well was removed, and the formazan product was solubilized in 150 μl of DMSO. The absorbance was measured at 570 nm by a Molecular Devices Spectra Max Plus automatic plate reader (Molecular Device, Sunnyvale, CA, USA) to determine cell viability.

### Cell treatment

HepG2 cells were starved in DMEM with 2% FBS for 9 h. To establish an *in vitro* model of diabesity, cells were stimulated with high glucose (36 mM glucose) and 0.25 mM OA for 12 h according to our previous study (14). Then, BB1-BB5 (50 and 100 μM) and Met (100 μM) were added for another 12 h. OA or BB1-BB5 was dissolved in DMSO and added to HepG2 cells in DMEM, and the final DMSO concentration received by the cells was 0.1% (v/v).

### Measurement of glucose uptake capacity

After the model was established, HepG2 cells were treated with BB1-BB5 (50 and 100 μM in low and high doses, respectively) for 12 h. 2-NBDG, a fluorescent deoxyglucose, was added to measure glucose uptake (15). The culture solution was discarded, and 2-NBDG (100 μM, diluted in DMEM without serum) was added and incubated again for 40 min. The culture solution was discarded and washed 3 times with PBS, and the fluorescence intensity was read at excitation filter 465 nm and emission filter 540 nm. In total, eight parallels were set up for each sample.

### Measurement of lipid accumulation

After the model was established, HepG2 cells were treated with BB1–BB5 (50 and 100 μM in low and high doses, respectively) in 6-well plates for 12 h. The culture solution was discarded, and 200 μl of RIPA lysis buffer was added after three washes with PBS and placed on a shaker for 10 min. The cell contents were homogenized and centrifuged (4°C, 10,000 rpm) to extract the supernatant. The TC and TG level in HepG2 cells were tested using the commercial kit by glycerol phosphate oxidase (GPO)-peroxidase (PAP) method based on several enzyme-driven reactions. The end product, hydrogen peroxide, which can be oxidatively coupled with 4-aminoantipyrine and phenol in the presence of peroxidase to yield a chromogen, was measured at 500 nm (16). In total, eight replicates were set for each sample.

### Oil-red-O staining

After the model was established, HepG2 cells were treated with BB1–BB5 (50 and 100 μM in low and high doses, respectively) in 6-well plates for 12 h. After treatment, in order to detect and quantify cellular lipid accumulation, cells were washed two times with PBS and fixed with 4% paraformaldehyde for 30 min in darkness. Subsequently, cells were stained with a freshly prepared working solution of oil-red-O for 30 min. Then, the cells were washed with 60% isopropanol solution for 5 min and observed under a microscope (Nikon, Tokyo, Japan).

### Statistical analysis

All data were expressed as the mean ± standard deviation (SD) of at least three independent experiments. The plots were obtained using GraphPad Prism version 8 (GraphPad Software, Inc., CA, USA). One-way analysis of variance (ANOVA) and Duncan's multiple range tests were performed to determine statistical differences between groups. Differences were considered statistically significant at a *p-*value of < 0.05.

## Results and discussion

### Isolation and identification of anthocyanins from blueberry

**Figure 1:**
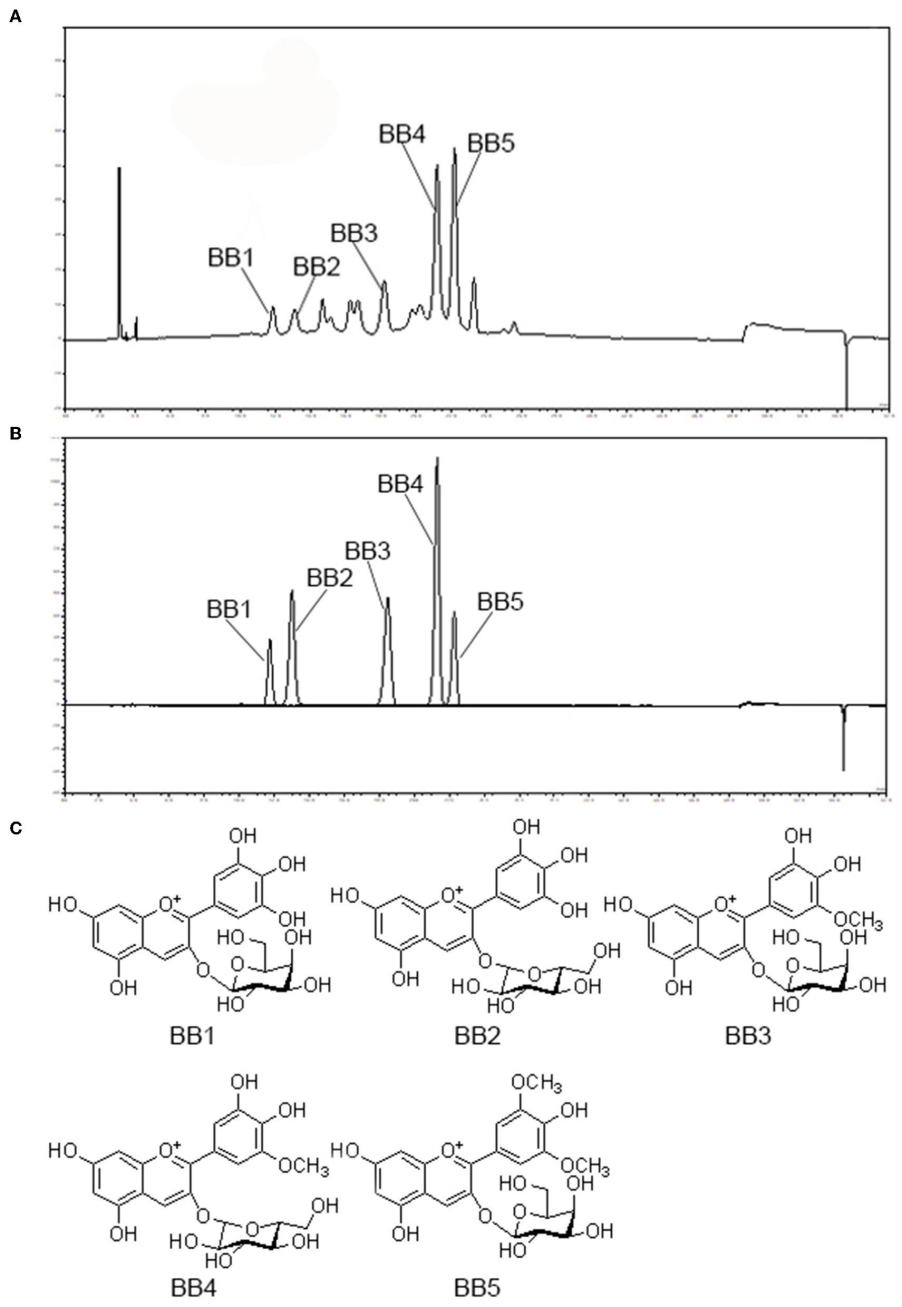
HPLC chromatogram of blueberry ethanol extract **(A)** and five anthocyanin compounds isolated from blueberry (BB1-BB5) detected at 520 nm **(B)** with their chemical structures **(C)**. BB1, delphinidin-3-*O*-galactoside; BB2, delphinidin-3-*O*-glucoside; BB3, petunidin-3-*O*-galactoside; BB4, petunidin-3-*O*-glucoside; BB5, malvidin-3-*O*-galactoside.

Five anthocyanins isolated from blueberry were identified by MS and NMR spectroscopic data as follows: delphinidin-3-*O*-galactoside (BB1), delphinidin-3-*O*-glucoside (BB2), petunidin-3-*O*-galactoside (BB3), petunidin-3-*O*-glucoside (BB4), and malvidin-3-*O*-galactoside (BB5), respectively (Figure 1).

### Antioxidant capacity

Oxidative stress is an imbalance between the prooxidant and antioxidant homeostasis that may lead to the development of oxidative damage. Diabesity patients usually show impaired antioxidant defenses and increased levels of inflammatory cytokines. Several studies have shown a strong association between the altered redox state, obesity, and diabetes (17). Antioxidants have been reported to reduce the risk of diabetes and obesity onset and improve some of the associated complications (18). A number of natural antioxidants have been tested for developing new therapeutic agents in the treatment of diabesity (19).

Various chemical tests have been used to evaluate antioxidant activity. Most of these tests are designed to measure the transfer of the hydrogen atom or electrons from antioxidants to different free radicals. In this study, the ORAC assay mainly evaluates the protection afforded by the test sample to a target molecule (FL) against oxidation by the peroxyl radicals induced by AAPH, and the scavenging capacity of DPPH and ABTS free radicals assay was used to evaluate the antioxidant capacity of blueberry anthocyanins. The results presented in Table 1, all five blueberry anthocyanins (BB1-BB5), show the scavenging power of both DPPH and ABTS^+^ free radicals, and their free radical scavenging concentration EC_50_ values were significantly lower than that of ascorbic acid. Among these compounds, malvidin-3-*O*-galactoside (BB5) possessed the highest scavenging capacity followed by delphinidin-3-*O*-galactoside (BB1). ORAC values varied from 9.44 to 13.23 μmol Trolox equivalent per μmol of anthocyanins. Delphinidin-3-*O*-galactoside (BB1) showed the highest antioxidant capacities followed by malvidin-3-*O*-galactoside (BB5). It seemed that malvidin-type anthocyanin had the strongest scavenging capability of DPPH and ABTS free radicals, followed by delphinidin-type and petunidin-type anthocyanins. Delphinidin-type and malvidin-type anthocyanins exhibited more significant oxygen radical absorbance capacity than petunidin-type anthocyanins. The anthocyanidin galactoside appeared to have stronger antioxidant capability than that of their glucoside. Previous research studies suggested that the high content of anthocyanins might be the major contributor to the antioxidant activity of blueberries (20). Our results confirmed that blueberry anthocyanins were the potential active compounds for the antioxidant property of blueberry. Further antioxidant tests in cell lines and even in animal models should be carried out to confirm their antioxidant capacity.

**Table 1:** DPPH and ABTS radicals scavenging activities of BB1-BB5.

**Compound**	**EC**_**50**_		**ORAC (μmol Trolox/μmol)**
	**DPPH (**μ**M)**	**ABTS (**μ**M)**	
BB1	39.91 ± 0.24^c^	19.01 ± 0.20^d^	13.23 ± 0.03^a^
BB2	61.37 ± 2.29^bc^	20.66 ± 0.67^cd^	9.44 ± 0.12^d^
BB3	63.16 ± 2.00^b^	24.20 ± 1.88^c^	11.31 ± 0.22^c^
BB4	62.11 ± 0.27^b^	39.26 ± 1.55^b^	11.02 ± 0.16^c^
BB5	38.83 ± 3.33^c^	14.32 ± 1.00^e^	11.89 ± 0.13^b^
ascorbic acid	112.19 ± 15.28^a^	44.45 ± 0.55^a^	——

All data are expressed as mean ± SD (n = 3). Different letters in each column denote a statistically significant difference at p < 0.05.

BB1, delphinidin-3-O-galactoside; BB2, delphinidin-3-O-glucoside; BB3, petunidin-3-O-galactoside; BB4, petunidin-3-O-glucoside; BB5, malvidin-3-O-galactoside.

### α-Glucosidase inhibition assay

α-Glucosidase digests the dietary carbohydrates and increases the postprandial glucose. Inhibiting this enzyme results in slower absorption of glucose and reduces postprandial glucose (21). In this study, five blueberry anthocyanins were tested for their inhibitory power of α-glucosidase *in vitro*. As shown in Table 2, five anthocyanins displayed potential in inhibiting α-glucosidase with IC_50_ from 68.33 to 218.2 μM. Delphinidin-3-*O*-galactoside (BB1) exhibited the strongest inhibitory activity followed by petunidin-3-*O*-galactoside (BB3), while their inhibitory activities were lower than acarbose. Results suggested that delphinidin-type and petunidin-type anthocyanins showed stronger α-glucosidase inhibitory activity than malvidin-type anthocyanins; in addition, the anthocyanidin galactoside showed a more remarkable effect than their glucoside. α-Glucosidase inhibitors, such as acarbose and miglitol, showed a reduction in postprandial glucose with an increase of insulin sensitivity in clinical studies and had been used in the treatment of type 2 diabetes and pre-diabetic states (21). The blueberry anthocyanins had the potential to inhibit α-glucosidase and may have a positive impact on glycemic control.

**Table 2:** Inhibitory activity of BB1-BB5 on α-glucosidase.

**Compound**	**IC_50_ (μM)**
BB1	68.33 ± 12.39^c^
BB2	239.7 ± 9.2^a^
BB3	68.54 ± 20.91^c^
BB4	218.2 ± 22.67^ab^
BB5	184.6 ± 10.91^b^
Acarbose	0.41 ± 0.02^d^

All data are expressed as mean ± SD (n = 3). Different letters in each column denote a statistically significant difference at p < 0.05.

BB1, delphinidin-3-O-galactoside; BB2, delphinidin-3-O-glucoside; BB3, petunidin-3-O-galactoside; BB4, petunidin-3-O-glucoside; BB5, malvidin-3-O-galactoside.

### Cytotoxic effects of BB1-BB5 on HepG2 cells

**Figure 2:**
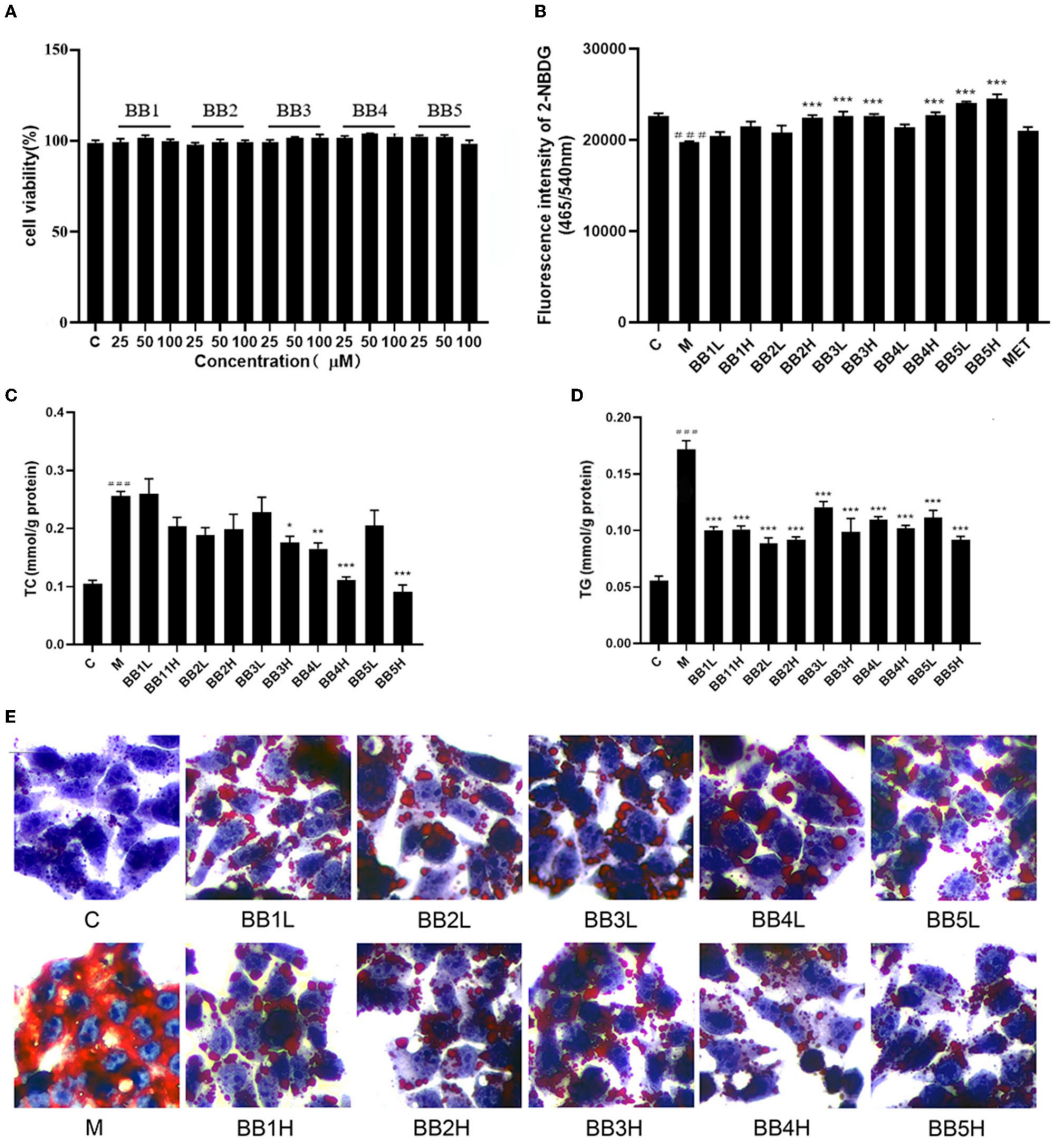
Effect of BB1-BB5 (25, 50, and 100 μM) on cell viability of HepG2 cells **(A)**. Cellular glucose uptake capacity by measuring the fluorescence intensity of 2-NBDG in HG-OA (36 mM glucose and 0.25 mM OA) stimulated HepG2 cells after treatment of low dosage (50μM, L) and high dosage (100 μM, H) of BB1-BB5, 100 μM of metformin (MET) was used as a positive control **(B)**. Low dosage (50 μM, L) and high dosage (100 μM, H) of BB1-BB5 alleviated lipid accumulation in HG-OA stimulated HepG2 cells. Levels of total cholesterol (TC) and triglycerides (TG) were measured **(C, D)**, and Oil-red-O staining was used to detect lipid droplets (200× magnification) in HG-OA stimulated HepG2 cells **(E)**. The results are the mean ± SD (*n* = 8). ^###^*p* < 0.001 *vs*. control group (C). ***p < 0.001, **p < 0.01 and *p < 0.05 *vs*. model group (M).

To avoid cytotoxicity, the survival rate of cells treated with different concentrations (25, 50, and 100 μM) of five anthocyanins (BB1-BB5) was determined by the MTT cell viability assay. As shown in Figure 2A, none of the five anthocyanins exhibited significant cytotoxicity compared with the control group.

### Glucose uptake assay

2-NBDG as a fluorescent glucose derivative can be used to assess the ability of cells to take up glucose (22). The fluorescence absorbance of HepG2 cells treated with high glucose and OA (HG-OA) was decreased by 12.7% compared to the blank control group, indicating a significant reduction (*p* < 0.001) in cellular glucose uptake capacity. A significant increase (*p* < 0.001) in fluorescence absorbance was observed after treatment with delphinidin-3-*O*-glucoside (BB2, 100 μM), petunidin-3-*O*-galactoside (BB3, 50 and 100 μM), petunidin-3-*O*-glucoside (BB4, 100 μM), and malvidin-3-*O*-galactoside (BB5, 50 and 100 μM) for 12h compared with HG-OA treated HepG2, suggesting remarkable recovery of cellular glucose uptake capacity (Figure 2B). Relatively speaking, petunidin-type and malvidin-type anthocyanins induced more notable glucose uptake activity than delphinidin-type anthocyanins.

It is well known that the liver plays a crucial role in metabolic homeostasis. It is the main place for the metabolism, synthesis, and redistribution of carbohydrates, glucose, and lipids. HepG2 cell is a human hepatocellular carcinoma cell line that retains many metabolic functions of hepatocytes, and the HepG2 cell line was commonly used as a model to study glucose uptake measurements (14). Many studies provided that the intake of blueberry is strongly associated with T2DM risk reduction (9). Blueberry treatment could decrease the blood glucose level and improve glucose tolerance in the animal model (23). In this study, four blueberry anthocyanins significantly restored the decreased glucose uptake capacity induced by high glucose and OA in HepG2 cells. These results suggested that blueberry anthocyanins might have a potential hypoglycemic effect by improving glucose uptake in the liver.

### Effect of BB1-BB5 on lipid accumulation

The liver is the central organ in the regulation of systemic lipid metabolism. The liver accrues fatty acids by hepatocellular uptake from the plasma and by *de novo* biosynthesis. Under normal circumstances, the liver stores only small amounts of fatty acids as triglycerides. During the progression of diabesity, hepatic carbohydrate and lipid biosynthesis become elevated, thus contributing to hyperglycemia and hypertriacylglycerolemia (24).

Oleic acid (OA) is an unsaturated fatty acid used to induce the dyslipidemia model in HepG2 cells. It is reported that OA exposure in cultured hepatocytes can evoke hepatic steatosis. OA-treated HepG2 cells were used for determining the effect of compounds on lipid metabolism and the underlying mechanism (23). In this study, 36 mM glucose and 0.25 mM OA induced a significant elevation in intracellular TC and TG levels (*p* < 0.001) in HepG2 cells compared with the control group. Incubation with petunidin-3-*O*-galactoside (BB3, 100 μM), petunidin-3-*O*-glucoside (BB4, 50 and 100μM), and malvidin-3-*O*-galactoside (BB5, 100 μM) for 12 h noticeably reduced the intracellular TC levels (*p* < 0.05, *p* < 0.01, *p* < 0.001, and *p* < 0.001) compared with the model group (Figure 2C). All five blueberry anthocyanin treatments induced a significant reduction in TG content (*p* < 0.001) at concentrations of 50 and 100 μM compared with the model group (Figure 2D). Moreover, to investigate whether blueberry anthocyanins influence intracellular lipid accumulation, oil-red-O staining was performed. High glucose and OA caused a prominent enhancement of lipid droplet accumulation in HepG2 cells compared with the control group. All blueberry anthocyanins notably reduced the accumulation of intracellular lipid droplets compared with the model group (Figure 2E). Comparatively speaking, malvidin-type and petunidin-type anthocyanins exhibited more significant hypolipidemic activity than delphinidin-type anthocyanins in HG-OA-treated HepG2 cells.

It was reported that a higher blueberry intake had the strongest association with less weight gain, lower fat mass, and less central adiposity in healthy individuals (25). Several studies confirmed that the consumption of anthocyanins-rich food prevents obesity in healthy subjects and improves obese individuals by reducing body weight and regulating metabolism and energy balance (26). In the present study, high glucose and OA induced remarkable elevation in intracellular TC and TG levels and significant lipid droplet accumulation in HepG2 cells. Blueberry anthocyanins treatment significantly reduced the intracellular TC and TG levels and inhibited the accumulation of lipid droplets in HepG2 cells. Results suggested that blueberry anthocyanins attenuated high glucose and OA-induced lipid accumulation in HepG2 cells, which may benefit the treatment of diabesity.

## Conclusion

In the present study, five anthocyanins were isolated and characterized from blueberry. Blueberry anthocyanins exhibited antioxidant power and inhibitory activity of α-glucosidase *in vitro*. Moreover, blueberry anthocyanins improved glucose uptake and attenuated lipid accumulation in high glucose and OA-induced HepG2 cells. All these results suggest that blueberry anthocyanins might benefit the treatment of diabesity by ameliorating antioxidant stress, controlling postprandial glucose, improving glucose uptake, and inhibiting lipid accumulation in the liver, while these functions remain to be confirmed in animal model experiments. Additionally, although some effects of anthocyanin structures on their pharmacological activities were discussed in this study, further structure-activity relationship needs to be investigated in the presence of more anthocyanin standards.

## Data availability statement

The original contributions presented in the study are included in the article/supplementary material, further inquiries can be directed to the corresponding authors.

## Author contributions

JC and WLL: study design. CWZ and HL: data collection and data analysis and drafting the manuscript. LLD, HC, JL, HFZ, and WLW: data interpretation. All authors take responsibility for the integrity of the data analysis and participated and approved the final version of the manuscript.
